# Cytotoxic CD4^+^ T cells in chronic viral infections and cancer

**DOI:** 10.3389/fimmu.2023.1271236

**Published:** 2023-10-25

**Authors:** Anna Malyshkina, Alicia Brüggemann, Annette Paschen, Ulf Dittmer

**Affiliations:** ^1^ Institute for Virology, University Hospital Essen, University of Duisburg-Essen, Essen, Germany; ^2^ Department of Dermatology, Venereology, and Allergology, University Hospital Essen, University of Duisburg-Essen, Essen, Germany

**Keywords:** CD4 T cells, CD4 CTL, cytotoxicity, chronic viral infections, cancer

## Abstract

CD4^+^ T cells play an important role in immune responses against pathogens and cancer cells. Although their main task is to provide help to other effector immune cells, a growing number of infections and cancer entities have been described in which CD4^+^ T cells exhibit direct effector functions against infected or transformed cells. The most important cell type in this context are cytotoxic CD4^+^ T cells (CD4^+^ CTL). In infectious diseases anti-viral CD4^+^ CTL are mainly found in chronic viral infections. Here, they often compensate for incomplete or exhausted CD8^+^ CTL responses. The induction of CD4^+^ CTL is counter-regulated by Tregs, most likely because they can be dangerous inducers of immunopathology. In viral infections, CD4^+^ CTL often kill via the Fas/FasL pathway, but they can also facilitate the exocytosis pathway of killing. Thus, they are very important effectors to keep persistent virus in check and guarantee host survival. In contrast to viral infections CD4^+^ CTL attracted attention as direct anti-tumor effectors in solid cancers only recently. Anti-tumor CD4^+^ CTL are defined by the expression of cytolytic markers and have been detected within the lymphocyte infiltrates of different human cancers. They kill tumor cells in an antigen-specific MHC class II-restricted manner not only by cytolysis but also by release of IFNγ. Thus, CD4^+^ CTL are interesting tools for cure approaches in chronic viral infections and cancer, but their potential to induce immunopathology has to be carefully taken into consideration.

## 1 Introduction

CD4^+^ T cells are essential players in immune defense and control of viral infections and cancer. These cells play a crucial role in providing cytokine signals and creating optimal conditions that facilitate the proper functioning of other immune cells such as macrophages, B cells and cytotoxic CD8^+^ T cells. Several distinct subsets of CD4^+^ T cells with diverse functions have been identified, including T helper (Th) cells 1 and 2, pro-inflammatory Th17 cells, follicular helper T cells, regulatory T cells (Tregs), and others, each characterized by their specific properties. However, in recent years, there has been a growing recognition of the direct protective effector role that CD4^+^ T cells can play in immune responses. Cytotoxic CD4^+^ T lymphocytes (CTL) were identified as an unconventional subset of CD4^+^ cells possessing cytotoxic capabilities that were thought to mainly attribute to CD8^+^ T cells.

First reports recognizing CD4^+^ T cells with cytotoxic potential appeared more than four decades ago (1), however, they were thought to be an artefact due to long-term culturing (2). Subsequent evidence has disproven this initial suggestion, demonstrating that antigen-specific CD4^+^ T cells exhibit direct cytotoxicity restricted by MHC class II molecules (3, 4). Since then, studies reporting on CD4^+^ CTL in both humans and animal species steadily increased in the context of viral infections and, recently, also in cancer.

Differentiation into effector CD4^+^ CTL involves the recognition of peptide antigens presented by MHC class II molecules on antigen presenting cells (APC) to naïve CD4^+^ T cells. In addition to this priming signal, naïve CD4^+^ T cells need at least two more signals for activation by APC: costimulatory molecules and pro-inflammatory cytokines (5). After antigen presentation, expression of the transcription factor Eomesodermin (Eomes) seems to be crucial for the development of cytotoxic T cells *in vivo* (6). Additionally, T-bet in cooperation with Eomes was suggested to modulate the cytotoxic program in CD4^+^ T cells (7, 8). T-bet and Blimp-1, induced by type I interferon and IL-2 signaling, were required for the generation of CD4^+^ CTL in influenza model (9, 10). However, other researchers also utilizing the influenza virus infection showed that Eomes, but not T-bet plays an important role in CD4^+^ CTL differentiation (11). Most likely both T-bet and Eomes are involved in CD4^+^ CTL fate, depending on their maturation stage. In fact, several studies demonstrated Eomes and T-bet co-expression in tumor-reactive CD4^+^ CTL, thereby emphasizing the link between cytotoxic and Th1 differentiation programs (7, 8, 12). Notably, a study by Śledzińska et al. in different tumor models demonstrated that cytotoxic features of tumor-reactive CD4^+^ Th1 cells can develop also independently of Eomes (13). In this study, depletion of Treg by anti-CTLA-4 treatment allowed for IL-2-dependent expression of Granzyme B in T-bet^+^ Eomes^-^ CD4^+^ T cells that was controlled by the transcriptional regulator Blimp-1. This suggest that initiation of the cytotoxic program in CD4^+^ T cells might be dependent on the immunological micromilieu and the pattern of their stimulation. Not only the presence of certain transcription factors is essential, but the absence of the others is also required. For instance, the T helper transcription factor, ThPOK, initially prompts the development of CD4^+^ Th fate and hinders thymocytes from maturing into CD8^+^ CTL (14). On the other hand, the Runx family member, Runx3, abrogates CD4 expression and supports cytotoxic lineage development (15). Although Runx3 was initially described to drive the cytotoxic program of CD8^+^ T cells, it has recently been demonstrated to be involved also in the development of CD4^+^ CTL (16). Not surprisingly, researchers showed that CD4^+^ CTL could be defined by the lack of the master regulator ThPOK, even though they originated from ThPOK^+^ progenitor cells (17, 18).

It is still a matter of debate whether CD4^+^ CTLs are a distinct phenotype or a CD4^+^ T cell subpopulation. Although researchers have tried to identify markers uniformly defining CD4^+^ CTL as well as their functional features, the characterization of these cytotoxic effectors remains challenging. In the past decades, CD4^+^ T cells have been distinguished into subsets solely based on the type of cytokines they produced (19–28). Considering the fact that the defined CD4^+^ T cell subsets are plastic and able to convert into other subsets, it has been proposed to define CD4^+^ T cell subsets based on both, their effector functions and phenotype (29, 30). More likely CD4^+^ T cells with cytotoxic activities develop from several differentiation pathways. Studies show that they can differentiate directly from naïve CD4^+^ T cells (10, 31). However, more often cytotoxic activities are acquired by already mature CD4^+^ T cells. Reports describe CD4^+^ cells with cytotoxic capacity arising from Th2 (32), Th17 (33), and even Tregs cells (34, 35), demonstrating the plasticity of CD4^+^ T cells. Nevertheless, the most common CD4^+^ CTL progenitor is a Th1-like subset expressing IFNγ alone or in combination with other cytokines and effector molecules (36–39). An early study by Qui et al. demonstrated that treatment of mice with agonistic anti-CD134 (OX40) and anti-CD137 (4-1BB) antibodies induced differentiation of naïve CD4^+^ T cells, both virus- and self-antigen specific, into cytotoxic effectors with Th1-associated cytokine production. The costimulation-induced Eomes expression and in addition IL-2 was required for the induction of cytotoxic features. Importantly, those costimulation-induced cytotoxic Th1 effectors showed anti-tumor activity in a murine melanoma model, confirming their cytotoxic activity *in vivo* (40).

What are the target cells for CD4^+^ CTL? Reports show that tumor cells and virus-infected cells can express MHC II and become targets for CD4^+^ CTL killing. B cells are infected by several chronic viruses and they constitutively express MHC II, since they are potential APC. But not only APC can become CD4^+^ CTL targets. Several factors can induce MHC II expression on cells that do not express these molecules under normal conditions. For instance, viral or bacterial infections can induce MHC II expression in lung epithelial cells via IFNγ signaling (41, 42). Additionally, epithelia or tumor cells were shown to express MHC II following irradiation or IFNγ treatment (43–46) and even constitutive MHC II expression in cancer cells has been described (see section 3.1). Subsequently, these cells become subject to CD4^+^ CTL-mediated killing. Moreover, several viruses evolved mechanisms to downregulate MHC I on infected host cells to evade CD8^+^-mediated killing (47, 48). As compensation, the infected host cells present viral antigens on MHC II, which allows elimination via MHC II-dependent pathways by CD4^+^ CTL. However, the frequency of these events *in vivo* is the matter of future investigations.

The direct cytotoxic mechanisms of effector CD4^+^ T cells are similar to those that are used by professional cytotoxic CD8^+^ T and NK cells. CD4^+^ CTL mainly utilize two effector mechanisms: granule-mediated exocytosis and death receptor-mediated pathways. The granule-mediated mechanism is exocytosis of specialized granules containing Perforin and Granzymes into target cells (39). Eomes, which was shown to drive expression of Perforin and Granzymes in CD8^+^ T cells, also plays a role in CD4^+^ T cell cytotoxicity (35). Death receptor-mediated pathways include Fas/FasL- or TRAIL-mediated apoptosis. Interaction of ligands, expressed on the effector CD4^+^ T cells, binds to its receptor on the target cells leading to recruitment of the death-inducing signaling complex and subsequently to apoptotic cell death (6, 49). Cytotoxic mechanisms of CD4^+^ CTL killing not only differ between virus infections or types of malignancy, but may be influenced by immunological factors even within one model system. For instance, IL-2 concentrations and the antigen dose controlled the switch between Perforin- or FasL-mediated cytotoxicity in the influenza virus model (10). In the FV model we also observed both FasL-mediated and exocytosis-mediated killing by CD4^+^ CTL, which was regulated by virus dose, infection phase, and application of immunotherapies (35, 50, 51).

CD4^+^ CTL often arise when CD8^+^ CTL are exhausted to partly compensate for their function. CD8^+^ CTL exhaustion occurs in many viral infections and malignancies, and is thoroughly described. CD4^+^ T cell exhaustion is less well studied, although some studies report on the exhaustion of conventional CD4^+^ T cells (Th1 or Th2 cells) during persistent infections (52–54). Very little is known about CD4^+^ CTL exhaustion. Using the FV model we showed that cytotoxic CD4^+^ T cells appear during chronic infection and keep persistent virus in check. They do this in the context of very profound CD8^+^ T cell exhaustion and Treg expansion (51, 55, 56). So there is obviously limited CD4^+^ CTL exhaustion during chronic FV infection. However, these CD4^+^ CTL kill via the Fas/FasL pathway and do not produce large amounts of cytotoxic molecules (50, 51). Similar to CD8^+^ CTL the exocytosis pathway of killing in CD4^+^ CTL is under suppression by Tregs during chronic infection. So CD4^+^ CTL are partially exhausted in persistent infections, but they can circumvent this by utilizing an alternative pathway for target cell lysis. Moreover, various studies indicated that CD4^+^ CTL responses in comparison to CD8^+^-mediated killing are more transient (57, 58). The differentiation of CD4^+^ T cells to CTL relies on constant antigen presentation, whether from a virus or a tumor, and ceases once the antigen level is reduced (57). Therefore, CD4^+^ CTL are most commonly reported from chronic or latent viral infections and tumor diseases, which is the focus of this review.

## 2 CD4^+^ CTL in chronic virus infections and virus-induced cancers

CD4^+^ T cells with a cytotoxic phenotype are only present as a small fraction under healthy physiological conditions. Their development is most likely restricted because they are potentially harmful as inducers of immunopathology. Their antigen recognition is not as precise as that of CD8^+^ CTL which increases the risk of unwanted cell killing. Even though the proportion of CD4^+^ CTL may increase in elderly individuals at least partly due to clonal expansion following repeated viral exposure (59, 60), the majority of studies on CD4^+^ CTL, as well as their initial investigation *in vivo*, originated from the realm of viral infections. Multiple researchers reported CD4^+^ CTL activity in acute influenza (9, 11, 61), ectromelia (62), vaccinia virus infection (63, 64). Such cells were also found in patients infected with mosquito-transmitted dengue (65) and West Nile (39) viruses. Recently, CD4^+^ CTL were described in individuals infected with SARS-CoV-2 (66). However, most reports come from chronic viral infections and CD4^+^ CTL have been identified in the blood of humans with cytomegalovirus (67), human immunodeficiency virus (HIV) (37, 68–71), and hepatitis viruses (72). CD4^+^ CTL from HIV-infected patients can kill HIV-infected target cells *in vitro* (73) and most importantly, cytotoxic CD4^+^ T cell responses are associated with disease outcome in HIV-infected patients (71, 74). This underscores an important physiological role for CD4^+^ CTL in controlling pathogens. CD4^+^ CTL have also been found in animal models of chronic viral infections, for instance, murine lymphocytic choriomeningitis virus (75), Friend virus (50), and simian immunodeficiency virus (76). The findings in these models and relevant human infections are summarized in this review.

### 2.1 Hepatitis viruses

Viral hepatitis is a significant global health issue, impacting hundreds of millions of individuals worldwide. Chronic hepatitis B, C, and D infections are strongly associated with liver cancer (77–79), as all three viruses infect hepatocytes. They are the reason that hepatocellular carcinoma (HCC) is the most frequently diagnosed malignancy in many regions worldwide (80). Despite notable advancements in treatment options against HBV (81), HCV (82), and HDV (83) in recent years, chronic viral hepatitis remain a wide-spread medical issue. Viral hepatitis, characterized by the persistence of the virus in the liver, is considered to be an immune-mediated disease, implying that the immune system plays a crucial role in the development and progression of chronic viral hepatitis (84), but also in virus control and resolvement of infection (85). However, our understanding of the mechanisms that regulate antiviral immunity during the chronic stage of hepatitis viruses remains insufficient.

**Figure 1:**
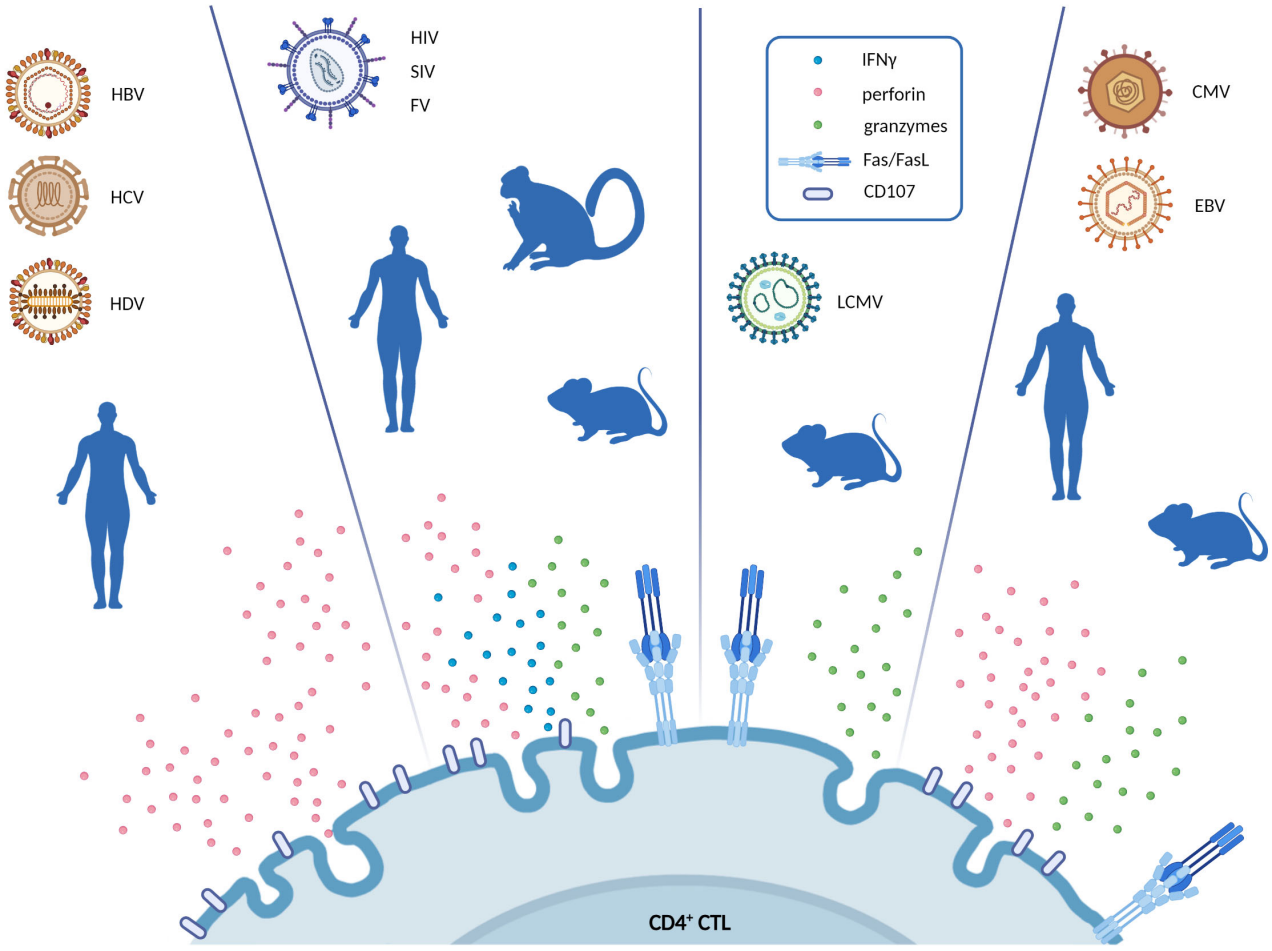
Schematic representation of the cytotoxic pathways exerted by CD4^+^ CTL in different chronic virus infections. For every virus infection, the blue silhouette represents the model in which CD4^+^ CTL were described (i.e., human, primate and mouse model). Figure highlights the main effector molecules secreted by CD4^+^ CTL in response to each virus (IFNγ, perforin, granzymes, CD107a, or FasL). Created with BioRender.com.

It is widely accepted that CD4^+^ Th cells are protective during HBV and HCV infections (86–89). At the same time the role of CD4^+^ CTL during hepatitis infection continues to be a topic of investigation. It has been shown that hepatocytes infected with hepatitis viruses express MHC Class II molecules on their surface (90) and acquire antigen presenting cell function (91). Thus, they could potentially be targeted by CD4^+^ CTL for killing. Indeed, CD4^+^ CTL, defined as Perforin-expressing CD4^+^ T cells, were detected in chronic viral hepatitis, especially in HDV infection (72) (**Figure 1**). Phenotypically, such CD4^+^ CTL exhibited a terminally differentiated effector phenotype (CD28^−^, CD27^−^) similar to that described for CD4^+^ CTL in other chronic viral infections (37). Even though direct cytotoxic killing of hepatocytes was not investigated *ex vivo*, authors provide indirect evidence that Perforin-expressing CD4^+^ CTL do kill infected cells and may accelerate fibrogenesis and hepatitis (92). Therefore, despite the fact that further studies are required to more precisely define the role of CD4^+^ CTL in viral hepatitis, this subset is very likely involved in immune-mediated pathology. For example, CD4^+^ CTL are known to mediated liver disease upon secondary infections with dengue virus (93). The authors of this study showed that dengue virus capsid-specific CD4^+^ CTL were responsible for liver cell killing through Fas/FasL interaction and also killed APC through Perforin expression (93). Thus, it is not surprising that MHC II-expressing hepatocytes infected with viruses may become targets of CD4^+^ CTL killing. Indeed, in the cohort of viral hepatitis patients there was a striking correlation between CD4 Perforin expression and aspartate aminotransferase levels that serves as a marker of hepatocyte damage (72).

On the other hand, the loss of CD4^+^ CTL in patients with HBV-mediated HCC was correlated with a higher mortality rate and a reduced survival time (94). Moreover, in tumor biopsies from the liver, CD107a degranulation marker (a surrogate marker for exocytosis in cytotoxic cells) on CD4^+^ CTL was significantly reduced in patients with an advanced cancer stage. This cytotoxic activity of CD4^+^ T cells was shown to be controlled by local Tregs (94). In a mouse model the beneficial therapeutic activity of CD4^+^ CTL was reported in a vaccination approach against HCC (95). In this study, tumor formation was controlled by vaccine-induced CD4^+^ T cells and this control was abrogated by anti-CD4 antibody administration. This preclinical model may suggest that CD4^+^ CTL inducing therapy in humans should be considered for further investigation.

### 2.2 Retroviruses

First evidence for the existence of CD4^+^ T cells with direct anti-viral effector functions came from studies of chronic retroviral infections in monkeys in the ‘90s (96). Since then, CD4^+^ CTL have been described in simian immunodeficiency virus (SIV) infection of rhesus macaques. Here, viremia increase, caused by CD8^+^ T cell depletion in SIV-infected macaques, was efficiently controlled due to a combination of antibody responses and expansion of circulating CD45RA^−^ CD28^+^ CD95^+^ CCR7^−^ Granzyme B (GzmB)^+^ SIV-specific CD4^+^ CTL (76). Similarly, Gag- and Nef-specific CD4^+^ T cell responses were found in CD8-depleted macaques during virus control (97). Another group demonstrated cytotoxicity of an SIV Gag-specific CD4^+^ T cell clone that had the capability to control viral replication (98). The main surface marker to characterize such CD4^+^ CTL in SIV-infected rhesus macaques was found to be CD29 (99, 100). CD29^hi^ GzmB^hi^ T-Bet^+^ gag-specific CD4^+^ T cells were also capable of shrinking the SIV reservoir during ART (99). SIV as well as HIV infects macrophages as well as CD4^+^ T cells and those constitutively express MHC II as APCs (101). In addition, activated CD4^+^ T cells start to express MHC II, and HLA-DR is even used as common activation marker for human CD4^+^ T cells (102). Thus, both cell types that propagate viral infection are potential targets for CD4^+^ CTL.

Similar to SIV, CD4^+^ CTL were found to be beneficial for people living with HIV. Despite the fact that CD4^+^ T cells are the main targets of HIV, including the crucial role of CD4^+^ T follicular helper cells in reservoir formation and maintenance (103, 104), studies support the vital role of CD4^+^ CTL in HIV control (70, 105). CD4^+^ CTL limit HIV pathogenesis in elite controllers (106). In acute HIV infection CD4^+^ CTL were characterized as GzmA^+^, IFN γ^+^, and CD40L^+^ and were associated with the reduction in viral loads (71). In another study similar observations were made and the phenotype of CD4^+^ CTL during acute HIV infection were described as Perforin^+^, GzmB^+^, and Eomes^+^ (70). Additionally, *ex vivo* studies revealed that virus-specific CD4^+^ CTL can kill HIV-infected macrophages and T cells (73). Moreover, an HIV vaccine candidate induced CD4^+^ CTL with lytic functions (107, 108). These lines of evidence suggest that CD4^+^ CTL play an important role in HIV control and therefore could be targeted as effectors in vaccine development and treatment interventions.

At the same time, inducing strong CD4^+^ T cell activation is a debatable issue in the field of HIV vaccination. It can be a double-edged sword since these cells may be favorable virus targets. In fact, it has been reported that GzmB^+^ cells harbored more HIV than GzmB^-^ cells in gut CD4^+^ T cells stimulated with enteric bacteria in the lamina propria aggregate culture model (109, 110). Nevertheless, the Thai HIV phase III prime-boost vaccination trial with ALVAC and AIDSVAX reported successful induction of HIV-specific CD4^+^ CTL (111). This vaccination regimen, combined a recombinant canarypox vector vaccine (ALVAC) and a recombinant glycoprotein 120 subunit vaccine (AIDSVAX), exhibited a moderate level of protection against HIV-1 infection partly correlating with the induction of polyfunctional effector CD4^+^ T cell responses (112, 113). Although stimulation of CD4^+^ CTL responses in HIV infection remains controversial, recent studies suggest that CD4^+^ CTL can compensate for reduced CD8^+^ T cell cytolytic activity against HIV in the setting of CD8^+^ T cell exhaustion (114), HIV-mediated downregulation of HLA I molecules (115), or CD8-associated HIV mutational escape (116). Therefore, while HIV-specific CD4^+^ CTL may be targeted by the virus and experience depletion during the early stages of infection, the remaining cells might play an important role in controlling viral loads (105).

#### 2.2.1 Friend virus

Friend virus (FV) was isolated from leukemic mice by Charlotte Friend (117) and has since been used for identifying genes that control susceptibility to retroviral infection. FV is a retroviral complex comprising Friend murine leukemia virus (F-MuLV), a replication competent helper virus that is nonpathogenic in adult mice, and spleen focus-forming virus (SFFV), a replication-defective virus responsible for pathogenesis (118). SFFV cannot produce its own particles because of large deletions in the gag gene and it spreads by being packaged in F-MuLV-encoded particles produced in cells co-infected by both viruses. Pathology in susceptible adult mice is characterized by a polyclonal proliferation and subsequent transformation of erythroid precursor cells, which results in gross splenomegaly. Resistant mice do not develop FV-induced disease because of their efficient immune response, but they are unable to completely clear virus-infected cells and therefore develop a life-long chronic infection. Although FV is also a retrovirus it has its own chapter here, because it is the retrovirus model system in which CD4^+^ CTL were studied in most detail among all retroviruses.

During the acute phase of FV infection, the primary and crucial role of CD4^+^ T cells is their helper function for the antibody responses and effector CD8^+^ T cells (55, 119, 120). The frequency of GzmB^+^ CD4^+^ T cells is extremely low during acute infection. We could previously show that during this stage of infection Tregs as well as CD8^+^ T cells negatively control the CD4^+^ CTL response against FV-infected cells as well as FV-transformed cells (51, 121), while the molecular mechanism of this suppression remains unknown. This cellular control is probably important to prevent CD4^+^ CTL induced immunopathology, which indeed occurs when GzmB^+^ CD4^+^ T cells are experimentally expanded by immunotherapy during an acute FV infection (122). However, when chronic FV develops, CD8^+^ T cells become functionally exhausted thereby allowing viral immune escape and the establishment of chronicity (123). Exhausted CD8^+^ T cells show a profoundly reduced killing capacity and have only limited anti-viral activity. We already showed in the late ‘90s that CD4^+^ CTL then take over in keeping virus replication in check (124). They do not induce pathology during chronic infection because antigen loads are low and CD4^+^ CTL numbers are too. The main reservoir of chronic FV is B cells, which are MHC II^+^ and therefore good targets for CD4^+^ CTL killing (125). The experimental proof that FV-specific CD4^+^ T cells develop cytotoxic activity during the chronic phase of FV infection against MHC II-expressing targets came from *in vitro* CTL assays (126) and subsequent *in vivo* CTL studies (50). The observed cytotoxic activity was FasL-dependent, while the exocytosis pathway and Granzyme production appeared to be suppressed by Tregs also in the context of chronic FV infection (50, 51). We have previously demonstrated that Tregs become highly activated and expand during an ongoing FV infection (51, 55, 56), so they constantly influence CD4^+^ T cell cytotoxicity. The established *in vivo* CD4^+^ CTL assay was used to quantify their killing capacity and defined which viral epitopes they recognize (50, 127). As expected, their killing potential was lower than that of FV-specific CD8^+^ CTL (128). Hence, these cytotoxic FasL^+^ CD4^+^ T cells can keep persistent FV in check and prevent viral rebound, but they are not capable of eliminating the viral reservoir.

Interestingly, also GzmB^+^ CD4^+^ T cells can be induced under certain conditions in the FV model. The cytotoxic activity of CD4^+^ T cells can be modulated by immunostimulatory therapies. The administration of agonistic antibodies that target the co-stimulatory molecule CD137 has been shown to trigger GzmB-dependent cytotoxic pathways in CD4^+^ T cells and makes them refractory to Treg-mediated suppression (50, 129, 130). We used this therapy to induce GzmB^+^ CD4^+^ CLT in chronically FV-infected mice, which were able to significantly reduce the viral reservoir size and even postponed viral rebound from the reservoir in the setting of a terminated anti-retroviral therapy (in press). Thus, CD4^+^ CTL might be interesting effector cells for shock and kill approaches in HIV cure studies.

### 2.3 Lymphocytic choriomeningitis virus

Another well-known murine model of persistent viral infection is the lymphocytic choriomeningitis virus (LCMV) infection. LCMV is a member of the Arenaviridae family and is commonly found in rodents, particularly mice. LCMV has been extensively studied to understand the mechanisms of persistent viral infections and immune responses. The virus infects a broad spectrum of cellular targets, including dendritic cells (131), macrophages (132), endothelial cells (133), epithelial cells (134), fibroblasts (135), and neurons (136). As professional APC, dendritic cells, and macrophages constitutively express MHS class II molecules on their surface and therefore can serve as CD4^+^ CTL targets. CD4^+^-mediated killing of target cells in LCMV-infected mice was reported in β2-microglobulin-deficient (β2m^−^) mice (137). Mice lacking β2m do not effectively express MHC class I complexes, resulting in a deficiency of CD8^+^ T cell responses (138). In this setting, LCMV-infected animals do not develop CD8^+^ cytotoxicity, but instead generate MHC class II-mediated CD4^+^ CTL (139, 140). These LCMV-specific CD4^+^ CTL express FasL and utilized the Fas-dependent killing pathway (141). However, other group showed that in LCMV infection both the FasL- and a Perforin-dependent pathway can contribute to CD4^+^ CTL killing (75). That again demonstrates that impaired CD8^+^ CTL responses are compensated *in vivo* by cytotoxic CD4^+^ T cells.

Other authors compared the *in vivo* killing mediated by CD4^+^ versus CD8^+^ T cells utilizing an *in vivo* CTL assay during LCMV infection (142). They detected substantial CD4^+^ CTL-mediated killing of target cells loaded with the immunodominant peptide LCMV-GP_64–80_ (143) in mice infected with LCMV that was measured at 16 hours after target cell infusion. This killing appeared less efficient as compared to the remarkably fast CD8-mediated *in vivo* killing of target cells, however researchers concluded that CD4^+^ and CD8^+^ CTL responses were similar in magnitude and were only slower due to the FasL-dependent pathway of CD4^+^ T cell killing (144). Additionally, a careful transcriptional investigation helped to discover a new marker for CD4^+^ CTL in LCMV infection (145). These cytotoxic cells express Eomes and GzmK together with uniquely high expression of the signaling lymphocytic activation molecule family member 7 (SLAMF7), a surface protein that was already described to characterize CD4^+^ T cells with cytotoxic potential in tumor (146) and autoimmune diseases (147).

Interestingly, experimental induction of CD4^+^ T cells during chronic LCMV caused lethal immunopathology in mice (148). Administration of vaccines to selectively induce CD4^+^ T cell responses resulted in severe generalized inflammation, a cytokine storm, and mortality. Furthermore, adoptive transfer of LCMV-specific CD4^+^ T cells following acute infection induced lethal inflammation (149). These results demonstrate the fine balance between anti-viral immunity and immunopathology for CD4^+^ CTL that has to be taken into account when designing immunotherapies or vaccines to induce such cells.

### 2.4 Herpesviruses

Viruses of the Herpesviridae family affect the majority of the human population. They establish lifelong infections, however are largely asymptomatic in healthy individuals, while causing severe disease in the immunocompromised hosts (150). CD4^+^ CTL were described in mouse and human herpesvirus infections. CD4^+^ T cells with cytotoxic effector functions were found in murine cytomegalovirus (MCMV) (151) and chronic infection of mice with γ-herpesvirus 68 (152, 153). Accordingly, CD4^+^ CTL were isolated from peripheral blood mononuclear cells of humans infected with human cytomegalovirus (HCMV) (36, 154). Other authors demonstrated HCMV-specific effector CD4^+^ T cells that expressed GzmA, GzmB, and Perforin with antiviral activity (67). Moreover, HCMV-pp65-specific CD4^+^ CTL were described in the cohort of older adults (155). CD4^+^ CTL have been observed in individuals infected with human herpesvirus-6B, suggesting their role in the long-term control of the disease (156).

The presence of CD4^+^ T cells exhibiting cytotoxic potential has also been identified in patients and mice infected with Epstein-Barr virus (EBV) (157). EBV is highly immunogenic virus which can be associated with the emergence of various types of cancer affecting B cells and epithelial cells (158). In individuals with infectious mononucleosis, blood samples reveal the presence of CD4^+^ T cells that express GzmB with potential anti-viral activity (159). CD4^+^ T cells isolated from tonsils, the hotspot of EBV infection, demonstrated cytotoxic potential *in vitro* (160). Cytotoxicity of EBV-specific CD4^+^ T cells was shown indirectly through the expansion of lytic cells from peripheral blood mononuclear cells, acquired from EBV-seropositive donors. The lytic activity was demonstrated to be facilitated through different pathways: the secretion of the cytotoxic molecules Perforin and Granzyme (161, 162) or via the Fas/FasL pathway (163). Moreover, lytic activity of EBNA1-specific CD4^+^ T cells against virus-transformed tumor cells was observed in all EBV-mediated malignancies, including Burkitt lymphoma (BL). BL cell lines serve as targets for the cytotoxic activity of CD4^+^ T cells specific against EBNA1 (164). In a mouse model, BL were eliminated in the absence of any CD8^+^ T lymphocytes, however no direct lytic CD4^+^ CTL activity could be detected in that model (165).

EBV is not only linked to the malignancies, but is associated with the development of autoimmune diseases, such as systemic lupus erythematosus (166), myasthenia gravis (167), multiple sclerosis (168), rheumatoid arthritis (169), celiac disease (170), and Sjögren’s syndrome (SS) (171). The development of SS has been linked to EBV infection, as salivary gland biopsies taken from SS patients demonstrate elevated levels of EBV DNA compared to healthy salivary glands. This suggests viral reactivation and an impaired immune system’s ability to control EBV latency (172). Interestingly, EBV-specific T cells show cross-reactivity to endogenous peptides from tears and saliva (173). While EBV is commonly found in salivary gland epithelial cells of healthy individuals, SS patients exhibit increased levels of HLA-DR expression in their salivary gland epithelial cells (172). This allows them to present EBV antigens to T cells and become targets of CD4^+^ CTL-mediated killing, which contributes to tissue damage. Indeed, a study investigating the cytotoxic immune response in ectopic lymphoid structures with persistent EBV infection in SS salivary glands revealed an increase in CD4^+^ GzmB^+^ CTL that was a risk factor for organ lesions (174). In contrast, CD8^+^ GzmB^+^ lymphocytes were impaired and did not correlate with the damage of the salivary glands. Moreover, a positive correlation has been observed between elevated levels of CD4^+^ CTL in the peripheral blood and their increased infiltration into the salivary glands, which is associated with disease progression and severity (175). Thus, CD4^+^ CTL might play an important role in the pathogenesis of EBV-induced disease.

Taken together, anti-viral CD4^+^ CTL are found in almost every chronic viral infection. They seem to compensate for CD8^+^ CTL responses when they are poorly induced or become exhausted. Their induction and differentiation are counter-regulated by Tregs, most likely because they can be dangerous inducers of immunopathology. CD4^+^ CTL often kill via the Fas/FasL pathway, but they can also facilitate the exocytosis pathway of killing. They are able to keep persistent virus in check, but especially their FasL pathway of killing is not sufficient to eliminate chronic viruses. CD4^+^ CTL are interesting tools for cure approaches in chronic viral infections, but their potential to induce immunopathology has to be carefully taken into account.

## 3 CD4^+^ CTL in solid cancers

Although CD4^+^ CTL have been recognized for decades in viral infections, they only recently attracted attention as direct anti-tumor effectors in solid cancers (176). Here we summarize preclinical and clinical data indicating killing of MHC class II-positive tumor cells by CD4^+^ CTL and highlighting the therapeutic potential of this T cell subset.

First evidences for direct anti-tumor activity of CD4^+^ CTL were provided by two studies in the murine melanoma model B16 in 2010 (43, 44). Tumor-bearing lymphopenic mice received adoptive therapy with CD4^+^ T cells expressing a transgenic T cell receptor (tgTCR) specific for the melanoma antigen TRP-1. The transferred T cells eliminated large tumors and mediated durable regression. Subsequent analyses showed production of IFNγ and GzmB- and Perforin-dependent killing of tumor cells by tgTCR CD4^+^ T cells in a MHC class II-dependent manner (43, 44). Combining T cell transfer with anti-CTLA-4 treatment enhanced the anti-tumor activity of CD4^+^ CTL (44). A third study later on demonstrated that melanoma control by TRP-1 tgTCR CD4^+^ CTL could also be improved when T cells were co-administered with an agonist antibody binding the costimulatory OX40 molecule (8).

Interestingly, evidence for the therapeutic efficacy of adoptively transferred CD4^+^ T cells in the clinical setting was provided in melanoma already in 2008. Durable remission of metastases was achieved upon treatment of a patient with *ex vivo* expanded autologous CD4^+^ T cell clones specific for the MHC Class II-restricted tumor antigen NY-ESO-1 (177). Those T cells secreted IFNγ upon antigen-specific activation and were detected over several months in the peripheral blood of the patient. Several years later, the Rosenberg team reported about a patient with metastatic cholangiocarcinoma who received treatment with *ex vivo* expanded autologous tumor infiltrating lymphocytes (TILs), containing CD4^+^ T cells specific for a MHC Class II-restricted mutant tumor antigen (neoantigen) (178). Partial regression of target lesions and disease stabilization were achieved by transfer of a TIL product, which contained around 25% of neoantigen-specific CD4^+^ T cells. Remarkably, upon disease progression the patient was retreated with TILs. In this case the TIL product contained >95% of neoantigen-specific CD4^+^ T cells, which again mediated disease regression (178). In a following study, patients with different cancers were treated with autologous CD4^+^ T cells purified from peripheral blood and engineered to express a TCR specific for the shared MHC Class II-restricted tumor antigen MAGE3. The tgTCR CD4^+^ T cells induced objective clinical responses, including a complete remission and partial regressions (179).

These cell therapy studies clearly demonstrated the clinical relevance of tumor antigen-specific CD4^+^ T cells in treatment of different cancers (177–179). Although data on cytolytic anti-tumor activity of the transferred CD4^+^ T cells was not provided, release of the effector cytokine IFNγ was demonstrated. Notably, in contrast to cytolytic granules, which act only locally at the T cell-tumor cell interface, IFNγ spreads into the tumor microenvironment (180). The cytokine triggers activation of the JAK1/2-STAT1 signaling pathways in bystander tumor cells, which can have cytostatic and cytotoxic effects (181, 182). Recent studies in different murine tumor models demonstrated that the long-distance IFNγ effects critically contribute to tumor control (183–185), and that control is lost when tumor cells acquire resistance to IFNγ, as we observed also in the clinical setting (182, 186). Thus, CD4^+^ CTL could kill their targets directly via cytolysis but also indirectly by IFNγ-dependent mechanisms. In fact, this has been demonstrated by a recent preclinical study in which mice with different tumor transplants, including melanoma, received adoptive therapy with chimeric antigen receptor (CAR)-modified CD4^+^ T cells. The transferred CAR-CD4^+^ T cells killed cancer cells via Perforin- and IFNγ-dependent mechanisms but could not eliminate IFNγ-resistant tumors (187).

Meanwhile, evidence for the presence of IFNγ-producing CD4^+^ CTL in solid human cancers have been generated by single cell RNA sequencing and flow cytometry analyses of tumors and tumor infiltrates. Based on the expression of Granzymes, Perforin, Granulysin and other cytolysis-associated markers, CD4^+^ CTL have been detected in bladder cancer, colorectal cancer, lung cancer, melanoma, and other tumors (146, 188–194). A study by Oh et al. in bladder cancer highlighted the presence of distinct subsets of CD4^+^ T CTL in the tumor microenvironment, expressing different combinations of cytolytic genes (*GZMA, GZMB, GZMK, PRF1)*. Approximately 50% of those CD4^+^ CTL were polyfunctional, showing concomitant expression of the effector cytokines IFNγ and TNFα (191). Important to note, MHC Class II-dependent CD4^+^ CTL were subjected to inhibition by tumor-resident Tregs (191).

Intense characterization of tumor antigen-specific CD4^+^ CTL was carried out also in melanoma. Cachot et al. applied antigen peptide-loaded multimers for isolation and subsequent characterization of NY-ESO-specific CD4^+^ CTL. Via this strategy, MHC Class II-restricted CD4^+^ CTL were detected *ex vivo* not only in tumors but also in tumor-infiltrated lymph nodes and peripheral blood of melanoma patients (146). Oliveira et al. analyzed in depth the antigen specificity and functional phenotype of tumor-resident CD4^+^ melanoma TILs. They found MHC class II-restricted neoantigen-specific cytotoxic CD4^+^ T cells largely exhausted and coexisting with MHC class II-restricted neoantigen-specific Treg (194).

So far, the indicated studies generated exciting data about the therapeutic potential and presence of CD4^+^ CTL in tumor infiltrates. But further intense investigations are needed to understand the molecular characteristics and development of tumor-specific CD4^+^ CTL in solid cancers in order to boost their anti-tumor activity. In this regard it should be mentioned that both, the bladder cancer and melanoma study showed elevated expression of SLAMF7 on tumor antigen-specific CD4^+^ CTL (146, 191) and that targeting SLAMF7 with agonistic antibodies enhanced the cytotoxic activity of CD4^+^ T cells (146). Thus, it is tempting to speculate that combining SLAMF7 agonists with personalized vaccines could be a promising strategy to specifically amplify cytotoxic anti-tumor CD4^+^ T cell responses.

### 3.1 Direct targeting of MHC class II-positive tumor cells by CD4^+^ CTL

Due to the fact that CD4^+^ CTL attracted attention in solid cancers only recently, there is still limited but growing data about their cytotoxic activity against tumor cells. So far, CD4^+^ T cell-mediated killing of either MHC Class II-matched or autologous tumor cells has been demonstrated in melanoma, bladder cancer and glioblastoma (12, 146, 191, 195, 196). Kitano et al. were the first to described cytolysis of human melanoma cells by NY-ESO-specific CD4^+^ T cells, that they found induced or enhanced in peripheral blood of patients treated with the anti-CTLA-4 blocking antibody ipilimumab. Those T cells expressed Perforin and GzmB and efficiently killed autologous melanoma cells in an MHC Class II-dependent manner (12). To achieve MHC Class II antigen presentation tumor cells were either transduced with CIITA the transcriptional activator of genes encoding the MHC Class II antigen presentation machinery (146, 197), or pretreated with IFNγ, known as potent inducer of MHC Class II expression (12).

Important to note, a sizable fraction of melanomas shows IFNγ-independent constitutive MHC class II surface expression (194, 195, 198–200), which in general is considered a specific feature of professional APC as dendritic cells, macrophages or B cells. So far, the mechanisms driving constitutive MHC class II expression in melanoma are poorly understood. Recently, we demonstrated that JAK1/2 signaling is involved in both IFNγ-induced and IFNγ-independent constitutive MHC Class II expression (195). The pathways triggering aberrant JAK1/2 activation in the absence of interferons remain to be determined. In line with this regulation, patient-derived JAK1/2-deficient melanoma cells displayed a stable MHC Class II-negative phenotype, resistant to CD4^+^ CTL (195). Interestingly, prior studies showed that ERK signaling negatively regulates constitutive but also IFNγ-induced CIITA expression (201, 202), suggesting that oncogenic Ras-RAF-MEK-ERK pathway activation in tumor cells counteracts MHC class II antigen presentation. So far, constitutive MHC class II expression has mainly been studied in melanoma, but seems to be present also in other cancers like glioma and lung cancer (195, 199, 203, 204), indicating a broader relevance of the MHC class II-positive tumor cell immunophenotype.

According to its role in CD4^+^ CTL activation, melanoma cell-intrinsic expression of MHC class II molecules has been associated with improved patient prognosis and response to immunotherapy with immune checkpoint blocking antibodies (200, 205). Similar data has been obtained for lung adenocarcinoma (206), but is lacking for most other tumors. This should encourage research to deepen our understanding on MHC class II regulation in different cancers as a basis for its therapeutic manipulation and killing of tumor cells by cytotoxic CD4^+^ CTL. As MHC class II-positive tumor cells can stimulate also tumor antigen-specific Treg (194), it is might be necessary to combine therapeutic MHC class II upregulation on cancer cells with Treg depleting strategies.

### 3.2 Virus-induced CD4^+^ CTL for therapy of solid cancers

**Figure 2:**
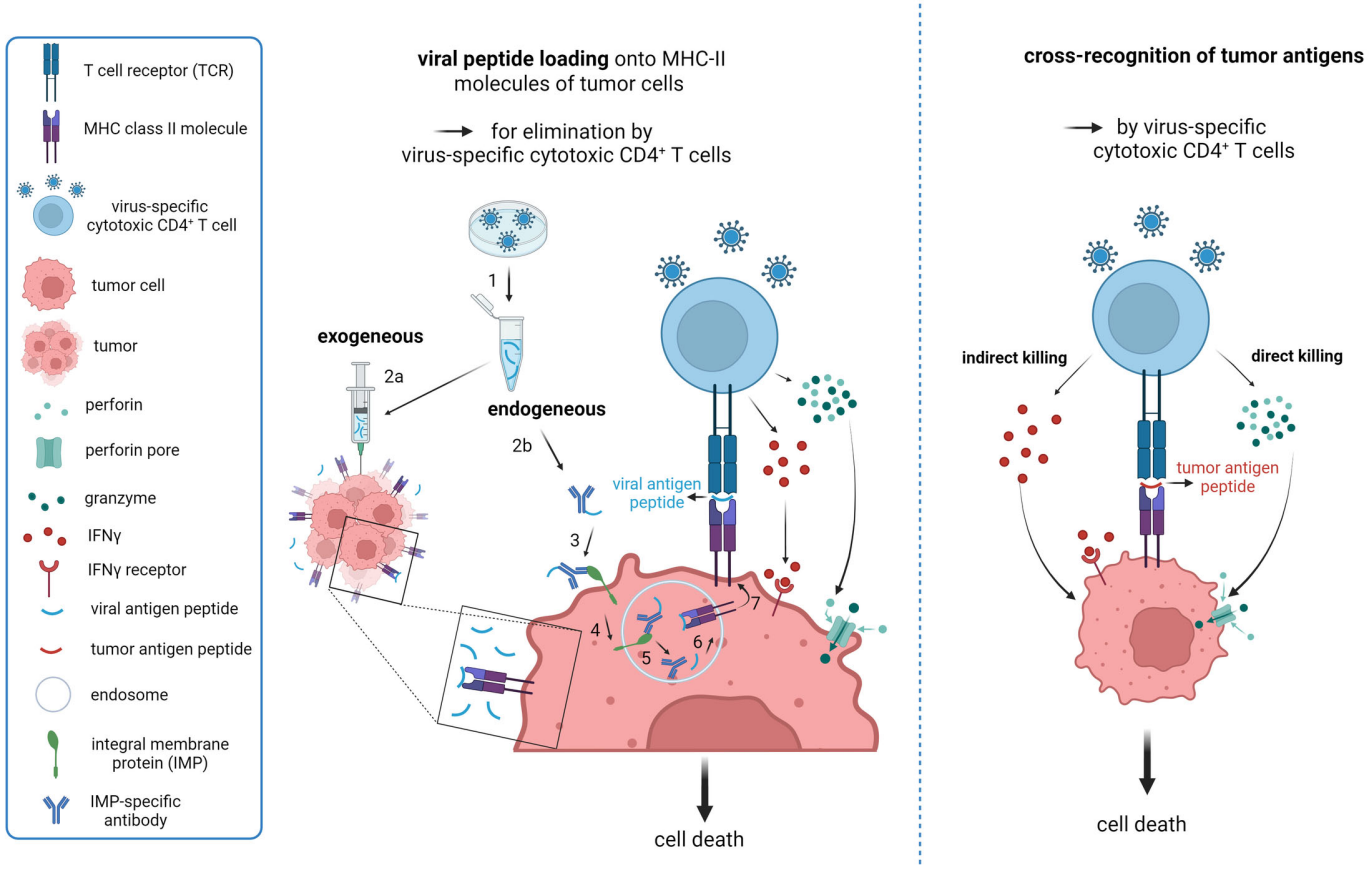
Strategies to exploit virus-induced cytotoxic CD4^+^ T cells in therapy of solid cancers. **(A)** Proposed model for reactivation of virus-specific CD4^+^ CTL based on endogenous and exogenous loading of viral-antigen peptides onto tumor MHC Class II molecules to mimic local re-infection with a previously encountered pathogen. 1. Synthesis of immunogenic peptides from chosen virus; 2a. Intratumoral injection of viral-peptide vaccine; 2b. Conjugation of immunogenic viral peptides to an antibody targeting a specific integral tumor membrane protein for internalization upon engagement. 3. Binding of the immunoconjugate to its target; 4. Engagement-triggered internalization of the immunoconjugate complex into the endosomal compartment; 5. Release of antibody from the complex and dissociation of the peptide from the antibody; 6. Loading of released viral peptide onto MHC Class II molecules; 7. Transport of the peptide-MHC complex to the cell surface for presentation to CD4^+^ T cells. The proposed endogenous loading model (2b-7) is based on work by Sefrin et al. (212). **(B)** Cross-recognition of MHC Class II-presented tumor antigen peptides by virus-induced CD4^+^ CTL based on sequence similarity. Potential killing-modes of CD4^+^ CTL are depicted. Created with BioRender.com.

As CD4^+^ CTL have been intensively studied in the context of viral infections, this led to the idea of exploiting those cells also in therapy on solid cancers. The concept is based on the observation that virus-specific T cells have been detected among the infiltrates of different cancers. For instance, TILs isolated from both lymph node and subcutaneous tumors of melanoma patients contained CD8^+^ T cells with specificity for viral antigen epitopes originating from CMV, EBV or influenza A (207). CD8^+^ T cells specific for epitopes from those viruses were present also among TILs from glioblastoma, colorectal and lung cancer (208, 209). In line with the clinical observations, a preclinical study in B16 melanoma demonstrated that virus-specific CD8^+^ T cells infiltrated cutaneous tumors not only upon acute infection with CMV or poxvirus, but were resident in lesions after poxvirus elimination and during the chronic state of CMV infection as well (210). The therapeutic potential of tumor-resident virus-specific memory T cells has already been demonstrated in different murine tumor transplant models. Activation of virus-induced T cells by intralesional injection of viral antigen peptides delayed tumor growth (209, 211) (**Figure 2A**). Alternatively, immunocojugates have been proposed for delivery of viral epitopes into tumors (**Figure 2A**). In this case, viral peptides were coupled to antibodies targeting a cell surface protein expressed on tumor cells. Upon immunoconjugate binding the surface complex was internalized and viral peptides were shuttled to the ER for loading onto MHC molecules. In a xenograft tumor model, systemic application of the immunoconjugates mediated recruitment of adoptively transferred virus-specific T cells into the tumor and combined administration of immunoconjugates with immune checkpoint blocking antibody suppressed tumor growth (212). Although the aforementioned studies focused on CD8^+^ T cells, it is expected that tumor infiltrates contain also virus-specific CD4^+^ CTL that could be exploited for therapy of solid cancers by applying similar therapeutic strategies.

Notably, activation of tumor-resident virus-specific T cells might even be applicable to cancer patients who not yet encountered an infection with the specific pathogen. Analyses of the CD4^+^ T cell repertoire from adults detected HIV-1-, HSV- and CMV-specific CD4^+^ T cells in blood from unexposed individuals (213). Surprisingly, these CD4^+^ T cells showed features of memory T cells even without direct antigen contact, namely expression of memory-associated genes, clonal expansion and rapid cytokine production. Further analyses on HIV-1-specific CD4^+^ T cells from unexposed individuals revealed TCR cross-reactivity towards similar environmental microbial peptides (213). Although this study did not specifically focus on cytotoxic CD4^+^ T cells, it provided important insights into cross-reactivity of virus-specific CD4^+^ T cells towards similar MHC class II-presented peptides.

Currently, T cell cross-reactivity towards viral and tumor antigens is under intense investigation, as referenced in (214) (**Figure 2B**). A recent study demonstrated very broad specificity of a MHC Class II-restricted CD4 TCR isolated form TILs of a glioblastoma patient. Those T cells recognized different peptides derived from pathogenic bacteria, commensal gut microbiota and also glioblastoma-associated tumor antigens (215).

Overall, these finding suggest that redirecting pathogen-specific CD4^+^ CTL towards tumor cells could be a promising mean to enhance the efficacy of immunotherapies. However, further investigations are needed to define the antigen cross-reactivity of CD4^+^ CTL, to develop approaches that recruit pathogen-specific, cytotoxic CD4^+^ T cells into tumors with low immunogenicity and to unleash strong cytotoxic T cell responses against tumor cells *in vivo* without causing severe immune-related adverse events.

## 4 Conclusion

Many reports on CD4^+^ CTL in chronic viral infections, virus-induced cancers, and virus-independent malignancies established the knowledge that CD4^+^ T cells not only serve as helper cells but also possess direct cytolytic activities, mainly in an MHC II-restricted way. Given that CD4^+^ CTL often play protective roles in antiviral or antitumor immunity, their molecular pathways of antigen control need to be investigated in detail and their possible detrimental effects should be studied when they are targeted for immunotherapy. Therefore, it is essential to define their mechanisms of cell differentiation and function as well as to describe their distinctive phenotypical markers. In order to modulate CD4^+^ CTL activity and improve antiviral and antitumor immunity, single-cell resolution approaches should be intensified to further deepen the characterization of this unique T cell subset in the future.

## Author contributions

AM: Writing – original draft, Visualization. AB: Writing – original draft, Visualization. AP: Writing – original draft, Writing – review and editing, Supervision. UD: Writing – original draft, Writing – review and editing, Supervision.
